# Life Cycle Assessment of Microbial 2,3-Butanediol Production from Brewer’s Spent Grain Modeled on Pinch Technology

**DOI:** 10.1021/acssuschemeng.3c00616

**Published:** 2023-05-22

**Authors:** Bikash Ranjan Tiwari, Rajarshi Bhar, Brajesh Kumar Dubey, Sunil K. Maity, Satinder Kaur Brar, Gopalakrishnan Kumar, Vinod Kumar

**Affiliations:** † Institut National de La Recherche Scientifique - Centre Eau Terre Environnement, Université Du Québec, Quebec City G1K9A9, Canada; ‡ Department of Civil Engineering, Indian Institute of Technology Kharagpur, Kharagpur 721302, West Bengal, India; § Department of Chemical Engineering, Indian Institute of Technology Hyderabad, Kandi, Sangareddy 502284 Telangana, India; ∥ Department of Civil Engineering, Lassonde School of Engineering, York University, North York, Toronto M3J1P3, Canada; ⊥ School of Civil and Environmental Engineering, Yonsei University, Seoul 03722, Republic of Korea; # School of Water, Energy and Environment, Cranfield University, Cranfield MK43 0AL, U.K.; ∇ Department of Biosciences and Bioengineering, Indian Institute of Technology Roorkee, Roorkee 247667 Uttarakhand, India

**Keywords:** 2,3-butanediol, brewer’s spent grain, global warming, life cycle assessment, sensitivity analysis, biorefinery

## Abstract

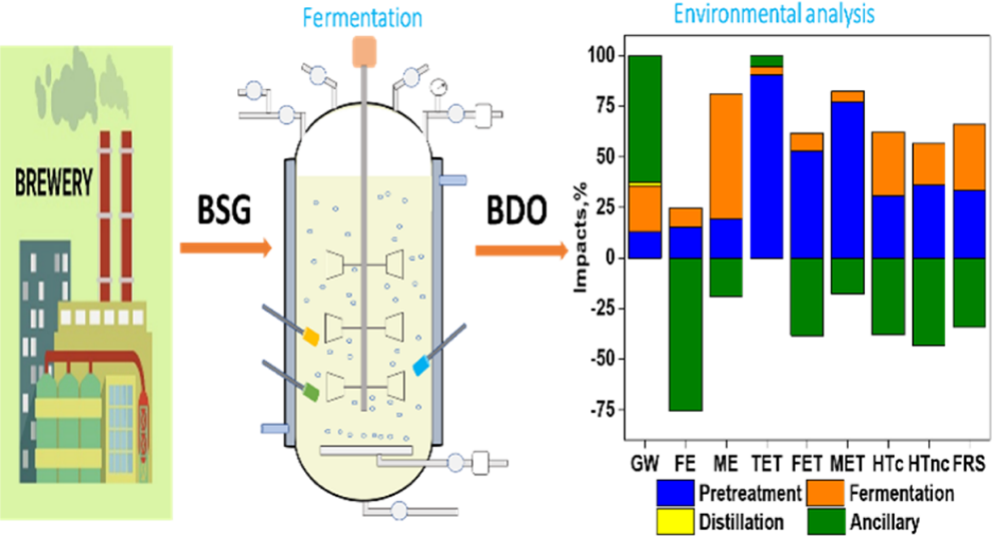

Microbial production of 2,3-butanediol (BDO) has received considerable attention as a promising alternate to fossil-derived BDO. In our previous work, BDO concentration >100 g/L was accumulated using brewer’s spent grain (BSG) via microbial routes which was followed by techno-economic analysis of the bioprocess. In the present work, a life cycle assessment (LCA) was conducted for BDO production from the fermentation of BSG to identify the associated environmental impacts. The LCA was based on an industrial-scale biorefinery processing of 100 metric tons BSG per day modeled using ASPEN plus integrated with pinch technology, a tool for achieving maximum thermal efficiency and heat recovery from the process. For the cradle-to-gate LCA, the functional unit of 1 kg of BDO production was selected. One-hundred-year global warming potential of 7.25 kg CO_2_/kg BDO was estimated while including biogenic carbon emission. The pretreatment stage followed by the cultivation and fermentation contributed to the maximum adverse impacts. Sensitivity analysis revealed that a reduction in electricity consumption and transportation and an increase in BDO yield could reduce the adverse impacts associated with microbial BDO production.

## Abstract 2

Environmental impact assessment for the bioproduction of 2,3-butanediol from brewer’s spent grains will aid industries in adopting a sustainable route for green BDO.

## Introduction

The world is heavily reliant on crude oil for the production of energy and chemicals. It is projected that the market for bio-based chemicals will reach around $128 billion with a market share of $14.5 billion for butanediol (BDO) alone.^1^ BDO is an emerging platform chemical widely used in cosmetics, food, pharmaceuticals, plasticizers, drugs, and softening agents.^1,2^ Notably, various BDO derivates, like 1,3-butadiene, acetoin, and methyl ethyl ketone (MEK), also possess high commercial value.^3−5^ Currently, BDO is commercially produced from fossil-derived crude oil.^6,7^ The crude oil is non-renewable, fossil in origin and its continued use pose environmental burden. Hence, increasing concern and awareness among market players regarding the impending environmental issues associated with large-scale fossil-derived platform chemicals have led to exploring sustainable bio-based production routes.

Microbial production of BDO using natural and genetically modified species such as *Enterobacter* sp., *Bacillus* sp., *Klebsiella* sp., *Saccharomyces* sp., *Paenibacillaceae* sp., and *Enterococcus* sp. has already been reported.^2,4,5^ The fermentative BDO production commonly utilizes 1G feedstocks (glucose) and 2G feedstocks such as corn stover, wheat straw, lignocellulosic biomass, etc. as substrates. However, an excellent choice of substrate would be an organic waste stream generated substantially from an agro-industrial sector which is either discarded or not exploited to its full potential. For instance, beer is one of the most consumed beverages in the world and brewer’s spent grain (BSG) is a major inevitable byproduct obtained during the brewing process. In 2016, EU-28 generated ∼10.8 million tons of nutrient-rich BSG with Germany and UK accounting for around 327,000 and 194,000 tons, respectively.^8,9^ Usually, a low economic value is associated with BSG, ∼USD 50 t^–1 ^ when utilized as an animal feed or biogas production, and the excess are disposed of in landfills.^10^ However, the challenge is to derive an economically more attractive product such as BDO as compared to current practices. Such an integrated biorefinery established with the low carbon manufacturing approach would be in-line with the principle of circular economy and maximize the gains of breweries. Subsequently, it will lead to effective waste management and promote environmental sustainability.

In our previous work, high-level BDO production (titer: 118.5 g/L; yield: 0.43 g/g; productivity: 1.65 g/L. h) was achieved using cellulosic fraction of BSG by mutant strain of *Enterobacter ludwigii* as the biocatalyst.^11^ This process was further subjected to techno-economic evaluation for a large-scale production in a centralized biorefinery with a BSG handling capacity of 100 metric tons per day.^9^ Hence, it is expected that the microbial BSG-derived BDO production via the microbial route has future industrial pertinence. Despite the demonstrated economic feasibility of industrial-scale BDO production from BSG, its environmental implications are yet to be ascertained. Previous studies have evaluated the environmental performance of BDO produced from different substrates such as oil palm empty bunches,^1^ 2G succinic acid,^12^ lignocellulosic biomass,^13^ and wheat straw.^14^ However, the environmental criteria are mostly limited to greenhouse gas (GHG) emission and energy consumption. Although GHG emission and energy perspectives are important environmental indicators, other significant impacts related to eutrophication, toxicity, resource depletion, etc. should also be estimated for a thorough sustainable bioeconomy approach.

Considering the available scope for evaluating the environmental performance of BDO production with a distinct 2G feedstock BSG, a comprehensive life cycle assessment (LCA) was conducted for the industrial scale process model handing 100 metric tons BSG per day. Since energy consumption in a biorefinery is one of the cost as well as pollution contributing parameters, a process integration tool for energy saving known as pinch technology was employed to enhance the thermal efficiency and effective utilization of heat within the process.^9^ As per the authors’ knowledge, this is the first study to conduct LCA for BDO production from BSG while incorporating pinch technology in process design. Furthermore, sensitivity and uncertainty analysis were conducted to identify hotspots within the BDO production process and evaluate potential impacts while the technology is still under the developmental phase. This study may aid stakeholders and policy makers to propose strategies for orienting future research toward a more sustainable and environmentally friendly outcome.

## Materials and Methods

### Process Chain

**Figure 1:**
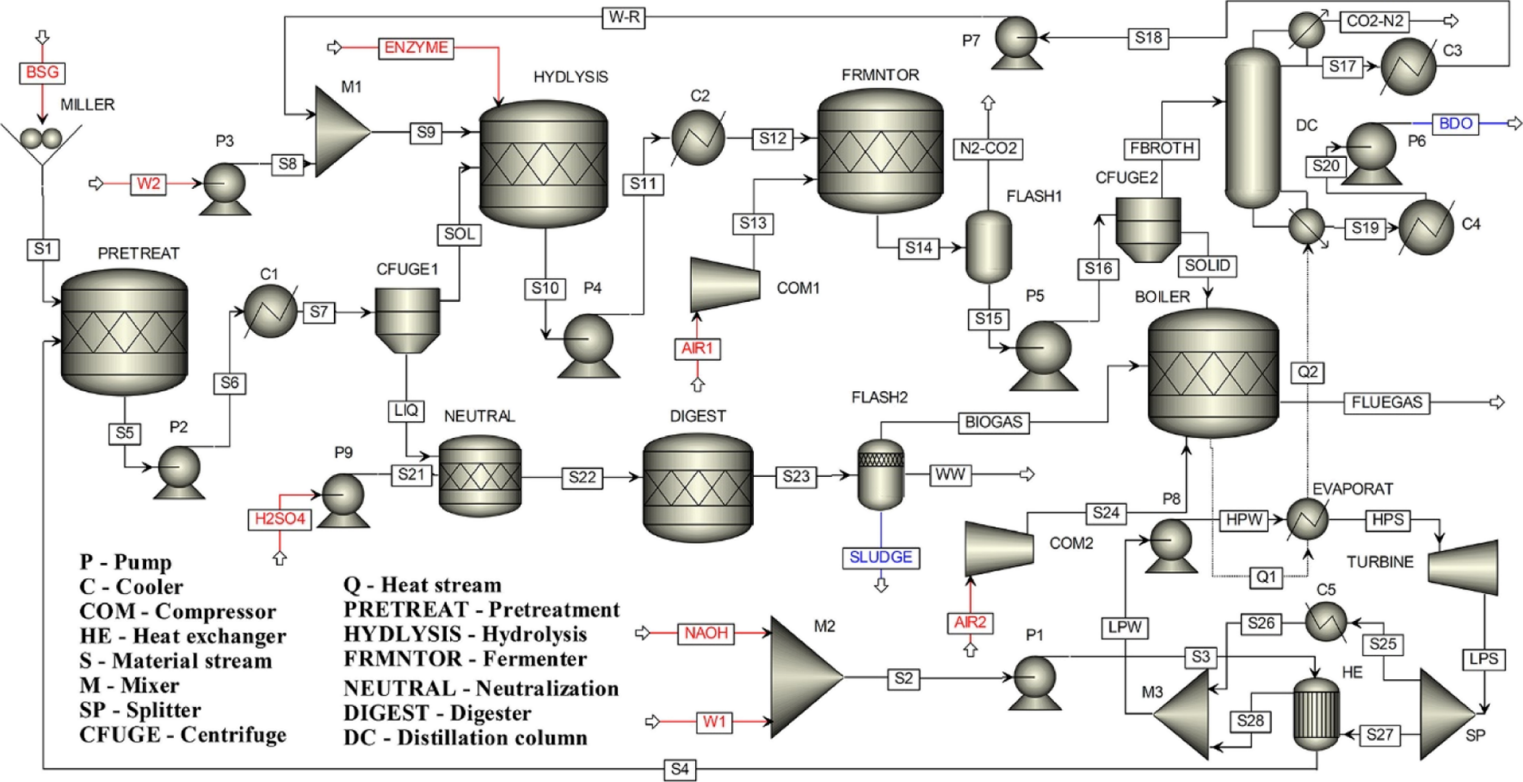
Process flow diagram of BDO production from BSG and modeled using pinch technology. Reproduced from Mailaram et al.^9^ Copyright 2022 American Chemical Society.

The LCA is based on a centralized biorefinery with a plant capacity of processing 100 metric tons BSG per day for BDO production based on our previous work including laboratory experiments^11^ and modeled in Aspen Plus V10.^9^ The annual BDO production capacity of the biorefinery is 5896.8 metric tons.^9^ The process model was divided into four sub-processes: pretreatment, fermentation, distillation, and ancillary processes. Prior to fermentation, the BSG was subjected to alkaline pretreatment and enzymatic hydrolysis. The hydrolysate from enzymatic hydrolysis was fermented using 10% (v/v) of the cultivated inoculum.^9^ Solid residues generated from fermentation and biogas generated from the anaerobic digester were used as fuel in the boiler to generate high-pressure steam. The fermented broth was thereafter distilled to obtain BDO. Detailed information regarding the processes can be found from the published works.^9,11^ The liquid side stream after alkali pretreatment was neutralized using H_2_SO_4_, prior to its anaerobic digestion (AD) to generate biogas (Figure 1). The subsequent wastewater generated was devoid of organic matter and only contains sodium sulfate.

**Figure 2:**
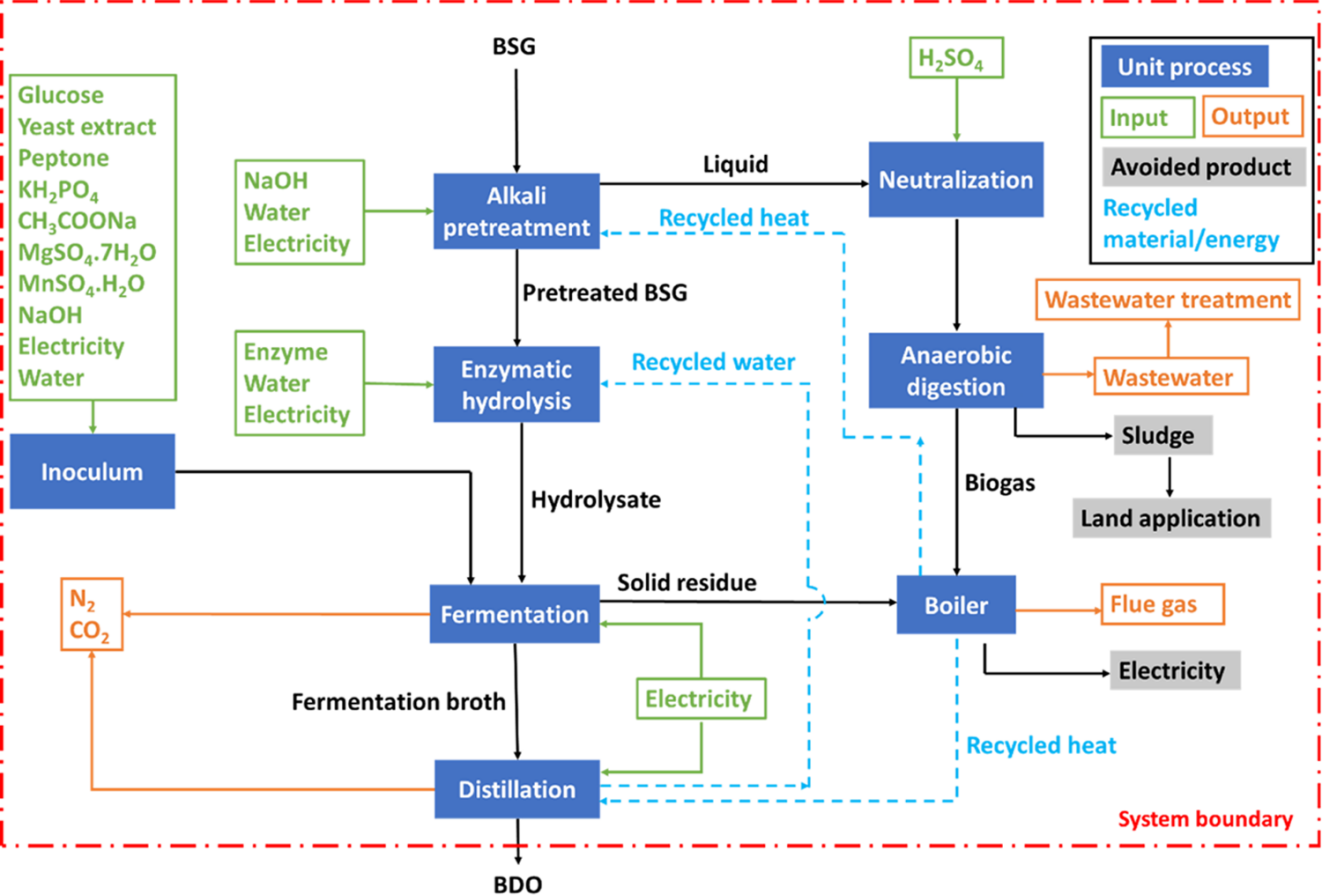
System boundary for LCA of BDO production from BSG.

It is important to note that the BDO production system considered in this study includes recycling of both water and process heat (Figure 2). The final waste generated was in the form of sludge and wastewater from AD, while gaseous emissions were released from boiler, fermentation, and distillation. The small amount of dissolved gases (CO_2_ and N_2_) in the fermentation broth was removed from the distillation column. The centralized biorefinery, however, involves the transportation of BSG far away from breweries. Hence, the BSG transportation was considered 500 km as the average distance of centralized processing plants from breweries. Similarly, it was assumed that the chemicals were also procured from a distance of 500 km. Since the sludge generated will be utilized for land application, the site of application of sludge derived fertilizer is also assumed to be within 500 km. Moreover, a zero-burden approach was associated with BSG used as a raw material for BDO production as BSG is not the primary product but a byproduct of the brewery.^15,16^

### Life Cycle Assessment

LCA is one of the most trusted tools for environmental assessment and comparison of different processes, operations or products.^17^ It is based on the guidelines provided by ISO 14040 and 14044 and consists of four major phases, namely, defining the goal and scope, life cycle inventory (LCI) analysis, life cycle impact assessment, and interpretation.

### Goal and Scope Definition

The goal of this LCA is to evaluate the environmental footprint of BDO production from BSG. As shown in Figure 2, the system boundary considered for BDO production is cradle-to-gate starting with the transportation of BSG generated in a brewery to the centralized BDO production facility. It includes BSG pretreatments followed by fermentation and distillation and subsequent AD and boiler for valorizing different waste streams. Based on the process goal, 1 kg BDO production was chosen as the functional unit for the LCA. The geographical scope of the study was limited to the United Kingdom (UK), and the LCA model was developed in SimaPro v9.1.0.

### Life Cycle Inventory

The LCI is representative of an industrial-scale BDO production unit, handling 100 metric tons BSG per day. Except the inoculum preparation phase which was adopted from our published work,^11^ the rest of the process inventory was based on process simulations in AspenPlus v10 software,^9^ as presented in Table 1. Also, these AspenPlus-simulated processes were integrated and modeled using pinch technology for an improved thermal efficiency and energy savings. The background footprint of the products and processes except enzyme considered in the LCI were adopted from the Ecoinvent database v3.7 (Supporting Information, Table S1). The environmental footprint of the “cellulase enzyme”, used during the enzymatic hydrolysis, was obtained from USLCI database.^18^

**Table 1:** LCI of BDO Production from BSG

	input			output		
	field	units	quantity	field	units	quantity
pretreatment	BSG	kg	6.19	separated liquid	kg	52.44
	electricity	kW h	0.25	hydrolysate	kg	13.19
	NaOH	kg	0.39			
	water	L	49.83			
	enzyme	kg	0.02			
	transportation^a^	t.km^d^	3.3			
fermentation	yeast extract	g	3.6	N_2_	kg	0.64
	glucose	g	14.48	CO_2_	kg	0.85
	peptone	g	7.24	fermentation broth	kg	10.23
	KH_2_PO_4_	g	2.9	solid residue	kg	2.26
	CH_3_COONa	g	7.24			
	MgSO_4·_7H_2_O	g	1.16			
	MnSO_4_·H_2_O	g	0.07			
	electricity	kW h	2.01			
	transportation^b^	t.km	0.18			
distillation	electricity	kW h	9.7 × 10^–4^	N_2_	kg	0.006
				CO_2_	kg	0.13
				BDO	kg	1
ancillary processes	H_2_SO_4_	kg	0.48	sludge	kg	0.673
	transportation^c^	t.km	0.57	electricity	kW h	2.39
	electricity	kW h	0.094	N-fertilizer (NH_3_)	kg	0.019
				P-fertilizer (P_2_O_5_)	kg	0.1
				K-fertilizer (K_2_O)	kg	0.01
				wastewater	kg	50.26
				CO_2_	kg	5.53
				N_2_	kg	17.69
				SO_2_	kg	0.04
				NO_2_	kg	0.64
				Na_2_SO_4_	kg	0.69
				electricity	kW h	2.39

a Refer to transportation of BSG.

b Chemicals for fermentation.

c Sludge for land application.

d t.km refers to tonne-kilometer.

Some of the major assumptions considered for compiling the LCI data are as follows:

- Density of the aqueous solution (with unconverted solids) after enzymatic hydrolysis is assumed as 1100 kg m^–3^ (1.1 kg L^–1^).
- The inoculum preparation was assumed to be near neutral pH condition.
- The power consumption by shaker during inoculum preparation was assumed as 0.23 W h kg _BDO_^–1^.^19^
- Per ton of sludge generated during AD was assumed equivalent to 27.55 kg of nitrogen, 32.63 kg of phosphorus, and 5.55 kg of potassium fertilizer.^20^
- The gaseous emissions due to application of 1 kg of sludge as fertilizer were 1.21 g of NH_3_, 0.15 g of N_2_O, and 0.15 g of NO_x_.^21^
- The raw BSG was subjected to drying in the breweries to reduce the cost associated with transportation.
- The wastewater generated during AD was assumed to have a specific gravity of 1.0.

### Life Cycle Impact Assessment

ReCiPe 2016 (H) was chosen as the life cycle impact assessment (LCIA) method for this study. It is one of the most frequently adopted LCIA methods, with footprint evaluation of both mid-point and end-point impacts.^22^ Some of the significant mid-point impact categories are global warming (GW), terrestrial acidification (TA), freshwater eutrophication (FE), marine eutrophication (ME), carcinogenic human toxicity (HTc), non-carcinogenic human toxicity (HTnc), terrestrial ecotoxicity (TET), freshwater ecotoxicity (FET), and marine ecotoxicity (MET). The sludge-based fertilizers and electricity are co-generated during the BDO production from BSG. This fertilizer could substitute the corresponding amount of commercially available fertilizers, hence reducing/avoiding the burden on the environment, which was attributed to virgin fertilizer production from the fertilizer industry. Similarly, the electricity generated could substitute a part of the electricity that would be generated from the grid and hence, avoid the corresponding impacts. The impacts have been allotted based on the “avoided burden approach”, by considering electricity generation and the application of the sludge as fertilizers to be ‘avoided burdens’.

### Sensitivity and Uncertainty Analysis

The overall environmental footprint and contributions of different phases may vary significantly with fluctuating input–output parameters of various critical aspects. These aspects differ from study to study and can be electricity, transportation distance, product and/or byproduct yields, energy generation, etc. The significance of such parameters is addressed via sensitivity analysis. In the present study, BDO titer, electricity consumption, and transportation distance were varied to observe the subsequent changes in the overall footprint and contributions from different processes/phases. In addition, Monte Carlo uncertainty analysis was performed to propagate the uncertainty linked to the BDO production process. The analysis was based on the lognormal distribution of the inventory data, and a pedigree matrix approach was considered where a scoring matrix was established based on data quality and accuracy. In this study, the pedigree matrix with data quality indicators, such as temporal, geographical, and further technological correlations, as well as reliability and completeness, was considered. These data with uncertainty values were subjected to Monte Carlo simulations within SimaPro software for a confidence interval of 95% extending up to 10,000 trials using the Recipe 2016 Midpoint (H).

## Results and Discussion

### Environmental Impacts Associated with BDO Production

The LCIA considers processes which are responsible for adverse impacts (expressed in +ve denominations) and also avoided products which lead to beneficial impacts (expressed in -ve denominations). The final impact value for each mid-point and end-point category is a cumulative of positive and negative impacts. Among the mid-point categories, emphasis is given to GW apart from eutrophication (freshwater and marine) and ecotoxicity (marine, terrestrial and freshwater, human carcinogenic, and non-carcinogenic) for their global relevance. The GW is usually considered a site generic category.^23^ The BDO production leads to a cumulative generation of 7.25 kg CO_2_ eq, where the major share of CO_2_ emission, i.e., 5.5 kg CO_2_ eq, is attributed to the combustion of solid residue generated from fermentation. Apart from this, GHG emission released from the fermentation of BSG hydrolysate, transportation (0.537 kg CO_2_ eq) and electricity consumption also contribute to GW. Carbon loss through gaseous emission at different stages of a biorefinery is a significant activity which generates adverse environmental impacts. Another study has also reported significant biomass carbon loss of around 20% (as CO_2_) in addition to protein recovery accounting for a loss of around 26% during the fermentation of distiller’s grain.^24^ Achieving better carbon utilization from biomass can lead to higher carbon credits and thus lower carbon emission. Similarly, employing approaches such as carbon capture and storage can reduce the carbon loss and eventually reduce the GW potential of the fermentation process.^25^ For example, flue gas can be utilized for microalgae cultivation for biodiesel generation, conversion of CO_2_ into bicarbonate, formate, and methanol.^26,27^ In addition, process improvement measures can also be undertaken to reduce carbon loss during fermentation.

In contrast to GW, eutrophication and toxicity vary with source location and hence site-dependent.^100^ Nearly half of the FE is attributed to use of NaOH and one-third to electricity along with enzyme used for enzymatic hydrolysis. However, the adverse impacts are overwhelmed by the beneficial impacts generated from substitution of AD sludge as fertilizers in agricultural lands, eventually resulting in FE of −1.18 ×10^–4^ kg P eq. ME was found to be 2.9 × 10^–5^ kg N eq where chemical consumption accounts for around 90% of the adverse impacts. It is noteworthy that glucose and yeast extract which are found to be environmentally benign as compared to other chemical inventories are the predominant factors along with NaOH. The same is true for TE where H_2_SO_4_ used for neutralization is the major contributor along with prominent contribution from electricity use, NaOH and transportation. The BDO production from BSG generated a net TET, FET, and MET of 11.17, 7.15 × 10^–4^, and 6 × 10^–3^ kg 1,4-DCB, respectively. It is evident that toxicity in the terrestrial ecosystem is considerably higher than marine and freshwater ecosystems. The transportation-related emission alone accounts for around 90% of the TET (11 kg 1,4-DCB). The rest of the TET impacts is attributed to chemical consumption (specifically NaOH), fuel combustion, and fossil-based electricity consumption. The same is also true for MET and FET, where transportation contributes to maximum adverse impacts in addition to other mentioned factors (1.6 × 10^–3^ kg 1,4-DCB and 7.8 × 10^–3^ kg 1,4-DCB). However, the contribution trend is different for toxicity in human where the BDO production generated net HTc and HTnc of 1.7 × 10^–3^ kg 1,4-DCB and 9.6 × 10^–2^ kg 1,4-DCB, respectively. Nearly half of the HTc is ascribed to the use of chemicals, notably NaOH and phosphoric acid. The other one-third is due to the electricity consumption related emissions, while the rest is shared between transportation (3.01 × 10^–4^ kg 1,4-DCB) and water consumed. On the contrary, electricity and transportation contribute equally to HTnc amounting for three-fourth of the impacts together while chemical consumption (mostly NaOH) account for nearly one-fifth of HTnc. Apart from these impact categories, resource consumption in the form of land use (LU), water consumption (WC), mineral scarcity (MRS), and fossil scarcity (FRS) are also considered in this study. While WC is primarily related to depletion of water in various activities, LU, MRS, and FRS are dominated by resource utilization for fossil-based electricity consumption. Moreover, yeast extract, WC, and transportation are responsible for nearly a quarter of LU, MRS, and FRS, respectively. In addition, both NaOH and H_2_SO_4_ are prominent factors contributing to MRS, FRS, and WC.

Apart from the mid-point indicators, ReCiPe allows evaluating the impacts in terms of damage to human health (Disability-Adjusted Life Years - DALY), ecosystems (species.yr), and resources ($) which are known as the end-point indicators.^28^ End-point indicators are vital as they can interpret the individual environmental flows mentioned in mid-point categories in terms of environmental relevance.^29^ Increase in temperature as a result of GHG emission is responsible for damage to human health as well as the ecosystem. As previously discussed for GW, apart from electricity and transportation, the emission from the combustion of solid residue leads to higher GHG emission which is allocated under an ancillary process. The damages to human health and ecosystems are estimated to be 7.7 × 10^–6^ DALY and 2.2 × 10^–8^ species.yr, respectively. The resources category ($0.0945) is attributed almost equally to the pretreatment and fermentation stage owing to the mineral loss due to chemical consumption and fossil loss caused by electricity and transportation demand.

### Contribution Analysis for Each Stage

**Figure 3:**
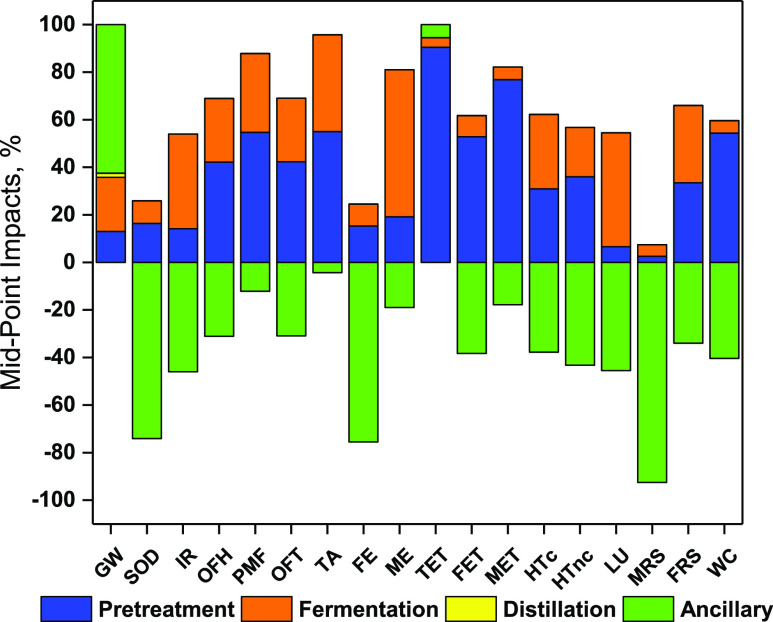
Mid-point impacts for 1 kg BDO production.

The pretreatment stage followed by the fermentation significantly contributed to all mid-point categories apart from GW where their cumulative share is around 36% (Figure 3). The pretreatment stage contributed heavily to TET, MET, and FET along with WC (86–93%) (Table 2). Similarly, the fermentation stage had a major role in ionizing radiation (IR), ME, and LU category (74–88%) where the contribution from pretreatment was limited to 12–26%. Moreover, both pretreatment and fermentation stage shared almost equal impacts for HTc and FRS. For the rest of the categories, pretreatment was the highest contributor in the range of 57–64%. A close look into pretreatment inventories revealed that the contribution of water and electricity was limited to WC and LU categories, respectively, while NaOH used for alkaline pretreatment and transportation of raw materials were responsible for the impacts attributed to all remaining categories. Transportation single handedly contributed to 88–96% of impacts attributed to TET, FET, and MET and LU (54%) during the pretreatment stage. For the pretreatment stage, chemical consumption is the major contributor (46–92%) for most of the midpoint categories except ecotoxicity, FRS, and WC. Electricity consumption was responsible for IR (34%) and LU (54%).

**Table 2:** Mid-Point Impacts for 1 kg BDO Production

impact category	unit	pretreatment	fermentation	distillation	ancillary	net Total
global warming	kg CO_2_ eq	9.37 × 10^–1^	1.65	1.28 × 10^–1^	4.51	7.23
stratospheric ozone depletion	kg CFC11 eq	9.23 × 10^–7^	5.35 × 10^–7^	1.82 × 10^–10^	–4.18 × 10^–6^	–2.72 × 10^–6^
ionizing radiation	kBq Co-60 eq	1.76 × 10^–2^	4.95 × 10^–2^	2.35 × 10^–5^	–5.72 × 10^–2^	9.92 × 10^–3^
ozone formation, Human health	kg NO*x* eq	2.12 × 10^–3^	1.35 × 10^–3^	5.94 × 10^–7^	–1.56 × 10^–3^	1.91 × 10^–3^
fine particulate matter formation	kg PM2.5 eq	1.07 × 10^–3^	6.48 × 10^–4^	2.67 × 10^–7^	–2.37 × 10^–4^	1.48 × 10^–3^
ozone formation, Terrestrial ecosystems	kg NO*x* eq	2.16 × 10^–3^	1.36 × 10^–3^	5.99 × 10^–7^	–1.58 × 10^–3^	1.94 × 10^–3^
TA	kg SO_2_ eq	2.70 × 10^–3^	2.00 × 10^–3^	8.03 × 10^–7^	–2.11 × 10^–4^	4.49 × 10^–3^
FE	kg P eq	3.64 × 10^–5^	2.19 × 10^–5^	8.88 × 10^–9^	–1.80 × 10^–4^	–1.22 × 10^–4^
ME	kg N eq	9.00 × 10^–6^	2.90 × 10^–5^	1.25 × 10^–9^	–8.92 × 10^–6^	2.91 × 10^–5^
TET	kg 1,4-DCB	9.66	4.36 × 10^–1^	1.44 × 10^–4^	5.84 × 10^–1^	1.07 × 10^1^
FET	kg 1,4-DCB	1.58 × 10^–3^	2.65 × 10^–4^	5.56 × 10^–8^	–1.14 × 10^–3^	7.05 × 10^–4^
MET	kg 1,4-DCB	7.06 × 10^–3^	4.94 × 10^–4^	1.58 × 10^–7^	–1.64 × 10^–3^	5.91 × 10^–3^
human carcinogenic toxicity	kg 1,4-DCB	1.89 × 10^–3^	1.91 × 10^–3^	5.96 × 10^–7^	–2.31 × 10^–3^	1.49 × 10^–3^
human non-carcinogenic toxicity	kg 1,4-DCB	2.10 × 10^–1^	1.21 × 10^–1^	6.21 × 10^–5^	–2.53 × 10^–1^	7.81 × 10^–2^
land use	m^2^a crop eq	1.89 × 10^–2^	1.37 × 10^–1^	3.96 × 10^–5^	–1.30 × 10^–1^	2.59 × 10^–2^
mineral resource scarcity	kg Cu eq	3.44 × 10^–4^	6.68 × 10^–4^	1.85 × 10^–7^	–1.25 × 10^–2^	–1.15 × 10^–2^
fossil resource scarcity	kg oil eq	2.84 × 10^–1^	2.76 × 10^–1^	1.22 × 10^–4^	–2.88 × 10^–1^	2.72 × 10^–1^
WC	m^3^	6.49 × 10^–2^	6.24 × 10^–3^	1.53 × 10^–6^	–4.82 × 10^–2^	2.29 × 10^–2^

However, in the fermentation stage, the electricity consumption for the operation of instruments was the highest contributor for all impact categories (>44%) except ME. The reason lies in the electricity supply mix for UK which is mostly dependent on fossil fuels (76.42%), followed by nuclear (13.67%), renewables (2.59%), and hydropower (2.54%) apart from 3.29% imported from France and Ireland (IEA 2011).^101^ This exploitation of fossil-based resources is directly associated with the adverse environmental impacts. Furthermore, impacts for ME were attributed to the consumption of glucose (60%) and yeast extract (30%) during inoculum preparation.

Impacts generated from ancillary activities are a cumulative of beneficial impacts associated with electricity generation and land application of sludge and adverse impacts owing to emissions, H_2_SO_4_ for neutralization, energy consumption, and wastewater treatment. Wastewater treatment generates adverse impacts for all categories except WC owing to the recovery of water. Electricity generation in boiler neutralizes the adverse demand generated by the electricity demand of the process for each impact category. The cumulative impact is beneficial for most of the impact categories in the ancillary activities stage (Figure 3). Especially for stratospheric ozone depletion (SOD), FE, and MRS, the land application of sludge to enrich the N, P, K generated enough beneficial impacts to overcome all their adverse impacts associated with BDO production. On the contrary, the cumulative impacts in this stage were in positive denominations for GW generated as a result of biogenic solid residue combustion in a boiler, and TET owing to the transportation of sludge to land (Table 2). Although the overall contribution to TET is 5%, ancillary activities account for highest contribution to GW, around 62% of the total GWP followed by fermentation (23%) and pretreatment (13%).

**Figure 4:**
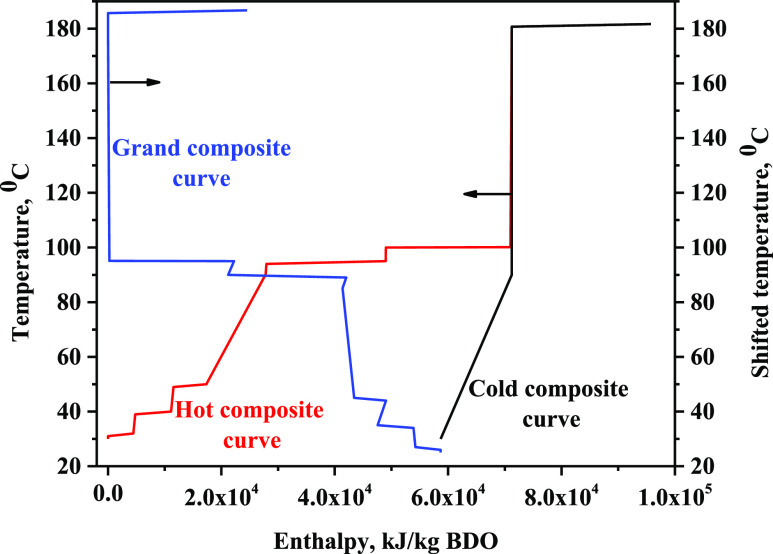
Temperature-enthalpy diagram. 100 metric tons BSG feed rate per day and 100 g/L BDO titer.

Distillation is one of the common separation techniques employed for the extraction of platform chemicals derived from fermentation where electricity consumption is the sole contributor to adverse impacts. Since BDO is a hydrophilic compound with a boiling point higher than water, the energy demand for the distillation process is very high.^30^ Rehman et al. reported that conventional distillation was the highest energy consuming process during the BDO production in the range of 58–66% of overall energy demand.^1^ This energy if derived from a fossil dominated electricity mix will further elevate adverse impacts. For example, high pressure steam utilized in the purification stage for BDO was found to be the second highest GW impact contributing process after cultivation with an emission of 0.61 kg CO_2_ eq kg^–1^ BDO.^13^ Hence, optimization of the distillation process and heat recovery from this unit is proposed to reduce the associated environmental impacts with energy consumption.^31^ In the present study, pinch technology was employed for the effective design of the heat exchanger network to minimize the external demand of energy and maximize the heat recovery. In the present study, pinch technology was employed for the effective design of the heat exchanger network to minimize the external demand of energy and maximize the process heat recovery, improving the thermal efficiency of the process. Temperature-enthalpy diagram with 10 °C as minimum temperature difference showed a shifted pinch point temperature of 175.7 °C, with minimum hot and cold utility demands of 24.5 and 58.7 MJ/kg BDO (Figure 4). These diagrams also showed a process heat exchange potential of 12.5 MJ/kg BDO. This efficient utilization of heat within the process is responsible for very low energy demand of the distillation process, i.e., the hot and cold utility demand of the process is reduced by 34 and 18%, respectively, accounting for an energy saving of 2.35 MW. This eventually translates to a low GW potential of 0.12 kg CO_2_ eq kg^–1^ BDO. Even for other impact categories contribution from the distillation stage was comparably less (Table 2). It was the least impact generating stage among all four.

### Sensitivity and Uncertainty Analysis

**Figure 5:**
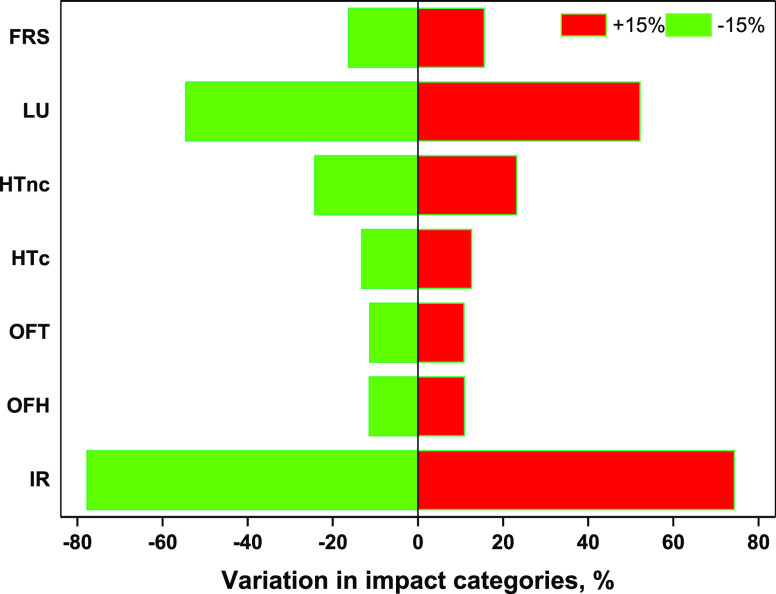
Sensitivity analysis for variation in electricity consumption by ±15%.

Among all the inventory listed for this LCA, electricity and transportation are two predominant sources leading to adverse environmental impacts for almost all categories. Hence, a sensitivity analysis was carried out for these inventories to ascertain the effect on environmental performance of the BDO production. Although the adverse impact of electricity consumption on environmental indicators is highest for the fermentation stage owing to the high energy demand, electrcity is a common input for all the four sub-processes involved in BDO production. Hence, the environmental performance of the BDO production was evaluated by varying the overall electricity consumption in the range of ±15%. The IR and LU were found to be most sensitive, where the impacts varied more than 70 and 52%, respectively. In addition, altering the electricity input by ±15 % led to a change in HTnc and FRS nearly 23 and 16%, respectively (Figure 5). The impact of change in input electricity was comparatively mild for ozone formation human health (OFH), ozone formation terrestrial ecosystems (OFT), and HTc, around 11–13%. The variation in GWP and eutrophication was less pronounced, around 3% for the change in electricity demand by ±15%. Still, it can be observed that reducing the electricity demand could bring about considerable reduction in environmental impacts.

**Figure 6:**
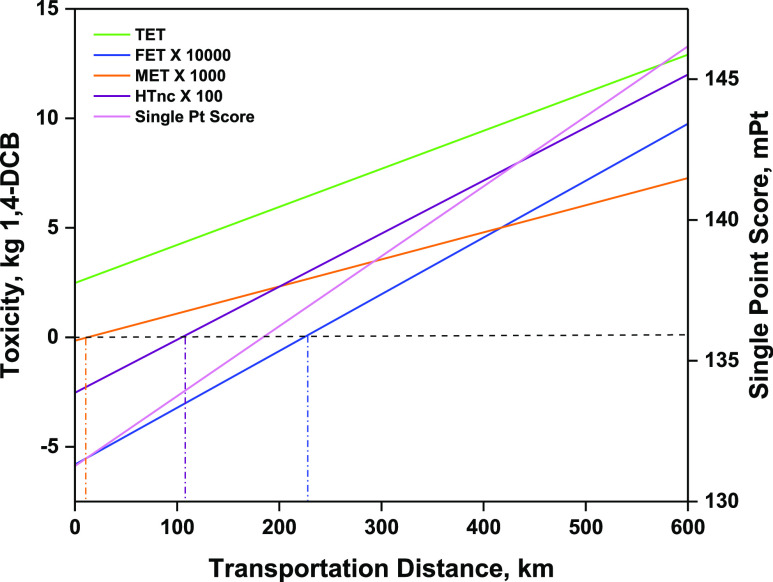
Sensitivity analysis for variation in BSG transportation distance.

Since transportation was the major activity contributing to adverse impacts in the categories of ecotoxicity and HTnc, hence, the distance of the biorefinery producing BDO from the brewery supplying BSG was considered for sensitivity analysis. Moreover, the single point score was also estimated at different transportation distances to gauge the effect of transportation distance on the overall impact of BDO production. The impacts are further proportionally reduced when the biorefinery is located closer than the base scenario of 500 km from the brewery. It was observed that with every 100 km reduction in distance, FET, HTnc, MET, and TET were reduced by nearly 26, 20, 17, and 13%, respectively. Moreover, when the biorefinery is located at a distance of 100 km, the adverse impacts in FET and HTnc are completely eliminated, while MET and TET are reduced by around 82 and 62%, respectively. To be precise, individual impacts for MET, HTnc, and FET will no more be harmful but beneficial to the environment at a transportation distance within 12, 105, and 225 km, respectively (Figure 6). Hence, for this case study, it is highly beneficial to locate the biorefinery within 12 km radius of the breweries. Considering a special case for a centralized biorefinery where the BDO production plant is located in close proximity to the brewery (∼1 km), the adverse impact for MET, HTnc, and FET is completely eliminated resulting in a beneficial scenario with a reduced single point score of 131.2 mPt.

**Figure 7:**
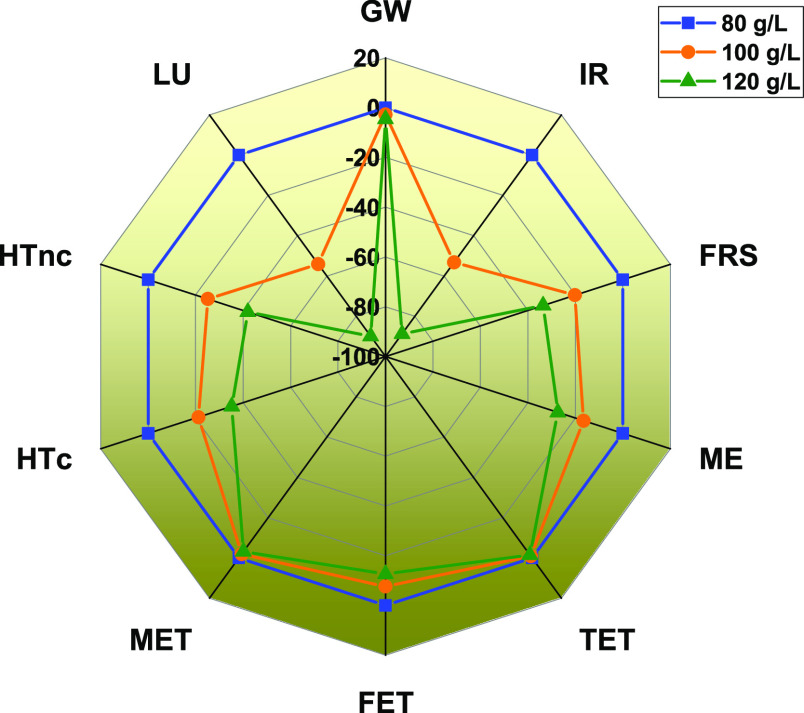
Variation in mid-point impacts with change in BDO titers.

It is observed that in addition to the strain efficiency, the titer also plays an important role in ascertaining the potential for industrial application of BDO production from BSG.^9^ As previously mentioned, a BDO titer of 100 gL^–1^ was considered in our process design. However, it has been reported that BDO accumulation as high as 150 gL^–1^ can be achieved through the biological route.^9^ Hence, a sensitivity analysis was carried out for a change in BDO titer in the range of ±20% to evaluate the corresponding change in environmental impacts for BDO production. With the increase in BDO titer from 80 to 100 gL^–1^, the per unit (per kg BDO) consumption of utilities in the system such as fermentation broth, chemicals, consumption of cooling water, and heat consumed in reboiler of columns reduces. The abatement of heat requirement of distillation column allows a higher fraction of heat generated in the boiler to be utilized for electricity generation. Moreover, the transportation related emissions and generation of flue gases also reduces. Subsequently, the adverse impacts of BDO production were found to reduce with the increase in titer from 80 to 120 gL^–1^ (Figure 7). As a result, impacts categories of IR and LU were significantly affected due to change in titers, around 75% reduction with the increase in BDO titer by 20% from 100 to 120 gL^–1^ (Figure 7). Similarly, the reduction in impacts was observed for HTnc (22%), HTc (18%), FRS (17%), ME (13%), and FET (8%). Although impacts reduced, the range was comparatively lower for GW, MET, and TET.

Uncertainty analysis is conducted to estimate the accuracy of the predicted results.^32^ Monte Carlo simulation is a complex process which uses sampling of data to generate random values repetitively which provide a probability-based estimation. The uncertainty analysis, via Monte Carlo simulations, was based on 10,000 simulation runs and 95% confidence interval (Table 3). Low CV-values were observed for FPM (11.8%), FRS (7.5%), GW (2.3%), LU (35.4%), ME (11.7%), MRS (24.3%), OFH (7.9%), OFT (7.8%), SOD (43%), TA (12.1%), and TET (4.5%).

**Table 3:** Monte Carlo Uncertainty Analysis for 1 kg BDO Production

impact category	unit	mean	median	SD	CV	2.50%	97.50%
fine particulate matter formation	kg PM2.5 eq	1.52 × 10^–3^	1.52 × 10^–3^	1.79 × 10^–4^	11.8	1.16 × 10^–3^	1.86 × 10^–3^
fossil resource scarcity	kg oil eq	2.80 × 10^–1^	2.79 × 10^–1^	2.09 × 10^–2^	7.5	2.40 × 10^–1^	3.22 × 10^–1^
FET	kg 1,4-DCB	3.81 × 10^–3^	2.71 × 10^–3^	5.19 × 10^–3^	136.3	–1.95 × 10^–3^	1.68 × 10^–2^
FE	kg P eq	1.05 × 10^–4^	6.80 × 10^–5^	1.64 × 10^–4^	155.6	–9.24 × 10^–5^	5.09 × 10^–4^
global warming	kg CO_2_ eq	7.25	7.25	1.70 × 10^–1^	2.3	6.92	7.60
human carcinogenic toxicity	kg 1,4-DCB	1.71 × 10^–2^	9.21 × 10^–3^	5.66 × 10^–2^	331.0	4.03 × 10^–3^	6.98 × 10^–2^
human non-carcinogenic toxicity	kg 1,4-DCB	2.58 × 10^–1^	2.15 × 10^–1^	2.95 × 10^–1^	114.3	–6.98 × 10^–2^	8.41 × 10^–1^
ionizing radiation	kBq Co-60 eq	1.23 × 10^–1^	6.80 × 10^–2^	2.00 × 10^–1^	163.4	1.59 × 10^–2^	5.47 × 10^–1^
land use	m^2^a crop eq	2.73 × 10^–2^	2.82 × 10^–2^	9.64 × 10^–3^	35.4	5.57 × 10^–3^	4.38 × 10^–2^
MET	kg 1,4-DCB	1.07 × 10^–2^	9.12 × 10^–3^	7.34 × 10^–3^	68.7	2.69 × 10^–3^	2.90 × 10^–2^
ME	kg N eq	4.83 × 10^–5^	4.80 × 10^–5^	5.68 × 10^–6^	11.7	3.83 × 10^–5^	6.06 × 10^–5^
mineral resource scarcity	kg Cu eq	–1.11 × 10^–2^	–1.08 × 10^–2^	2.71 × 10^–3^	–24.3	–1.75 × 10^–2^	–6.86 × 10^–3^
ozone formation, Human health	kg NO*x* eq	1.95 × 10^–3^	1.95 × 10^–3^	1.53 × 10^–4^	7.9	1.64 × 10^–3^	2.25 × 10^–3^
ozone formation, Terrestrial ecosystems	kg NO*x* eq	1.98 × 10^–3^	1.98 × 10^–3^	1.55 × 10^–4^	7.8	1.68 × 10^–3^	2.29 × 10^–3^
stratospheric ozone depletion	kg CFC11 eq	–2.82 × 10^–6^	–2.66 × 10^–6^	1.21 × 10^–6^	–43.0	–5.66 × 10^–6^	–9.44 × 10^–7^
TA	kg SO_2_ eq	4.58 × 10^–3^	4.58 × 10^–3^	5.56 × 10^–4^	12.1	3.50 × 10^–3^	5.66 × 10^–3^
TET	kg 1,4-DCB	1.12 × 10^1^	1.12 × 10^1^	5.09 × 10^–1^	4.5	1.03 × 10^1^	1.22 × 10^1^
WC	m^3^	2.20 × 10^–2^	3.10 × 10^–2^	1.72 × 10^–1^	779.4	–3.37 × 10^–1^	3.43 × 10^–1^

### Comparison with Other LCA Studies for BDO Production

Due to fewer studies on environmental analysis of 2,3-BDO (or BDO as we abbreviated), the present study also compares the environmental footprint associated with the other isomeric form, i.e., 1,4-BDO. Though they have been considered as alternatives, it is important to highlight that 2,3-BDO and 1,4-BDO are two different chemicals with varying physicochemical properties as well as end applications. 2,3-BDO is commonly used as a blending agent, crosslinking agent for specific hard-rubber materials, solvent for dyes, and in resins. On the other hand, 1,4-BDO is frequently used as humectants, monomers for resins, chemical intermediates for plasticizers, tetrahydrofuran, and resins. The difference in impacts is a culmination of factors such as variation in feedstock, location, processes, system boundary considerations, titers, etc. All the studies except Forte et al. have estimated only CO_2_ emission in contrast to the present study where a more comprehensive analysis with more environmental categories is performed.^14^

Rehman et al. reported that oil palm farming integrated with a biorefinery will lead to 6.8 kg CO_2_ benefits per kg BDO while considering the key material inputs only.^1^ However, the present study incorporates a comparatively holistic system boundary also including cultivation and growth of microbes, transportation, as well as wastewater treatment. Similarly, a comparison of BDO production from 2G succinic acid and direct C6 sugar fermentation revealed a GW of 2.05–2.37 and 0.16–0.54 kg CO_2_ eq where embedded biogenic carbon is considered as negative emissions.^12^ On the same lines, considering GHG emission during fermentation and the carbon sequestered in the fermentation solid residue as biogenic will further reduce the GW by 6.35 kg CO_2_ eq for this study and the subsequent GHG emission will be comparable to Patel et al.^1,2^

BDO production from cardoon lignocellulosic biomass^13^ estimated a total emission of 2.82 kg CO_2_ eq, where 1.94 kg of CO_2_ eq was attributed to the cultivation phase, while the biorefinery phase contributed 0.813 kg CO_2_ eq. Since the substrate was a lignocellulosic feedstock where significant inventory is utilized for its growth, the cultivation phase was the highest contributor to carbon footprint. However, the CO_2_ captured during the biomass growth was not included in the impacts. The transportation accounted for a very small fraction of the total GHG emission (0.067 kg CO_2_ eq) since the transportation distance of 100 km (return) was considered from the field to the biorefinery. In the present study, the transportation distance from brewery to biorefinery was considered to be 500 km which leads to higher contribution of 0.53 kg CO_2_ eq. Furthermore, the high pressure steam for the distillation column reboiler leads to a comparably higher impact of 0.61 kg CO_2_ eq for Bari et al.^13^ since it is derived from an external fossil source, whereas in the present study, the solid residual biomass received after fermentation and biogas were combusted by air in the boiler to generate high-pressure steam.

A cradle to factory gate LCA based on a renewable source, i.e., wheat straw for BDO production reported impacts, which were lower than the fossil-based BDO production.^14^ Energy requirement was supplemented by the combustion of unconverted solids in a CHP plant. Moreover, the CO_2_ emission from the CHP plant was considered biogenic in origin cumulatively around 5 kg biogenic CO_2_ per kg BDO. In the present study CO_2_ emission from all possible sources such as fermentation, distillation, AD and wastewater treatment were considered without excluding those biogenic in origin. As mentioned earlier, eliminating the biogenic CO_2_ stored in the solid residue will result in reducing the final emission to around 0.9 kg CO_2_ eq which is considerably lower than reported by Forte et al.^14^ A base scenario was considered for the production of fossil based 1,4-BDO retrieved from the Ecoinvent database. Following the same trend of comparison, it was observed that impacts in all the 14 categories ME, TET, MET, and LU are lower than the fossil based BDO production when biogenic carbon is excluded. Nevertheless, it should be noted that there are no adverse impacts resulting from BDO production in the present study for the categories of SOD, FE, and MRS.

## Conclusions

The LCA identified electricity consumption and transportation to be major impact generating activities. Impacts associated with distillation were very low owing to the improved process design using pinch technology leading to energy savings. Global warming impacts are comparable to other studies when biogenic carbon is considered having zero impacts. Achieving better carbon conversion from biomass and employing carbon capture and storage can further reduce the GW potential. Sensitivity analysis revealed that the reduction in electricity consumption and transportation distance in addition to the increase in BDO titers can make the process more environmentally friendly. This study can aid the stake holder in a better decision making for a future biorefinery to achieve environmental sustainability.

## Supporting Information Available

Table of Inventory for background processes used in life cycle analysis (PDF)

## Abbreviations

BDO: 2,3-butanediol
BSG: brewer’s spent grain
CHP: combined heat and power
CF: cultivation and fermentation
DALY: disability-adjusted life years
DCB: dichlorobenzene
FRS -: fossil resource scarcity
FE -: freshwater eutrophication
FET -: freshwater ecotoxicity
FU: functional unit
GHG: greenhouse gas
GW: global warming
HTc: human toxicity: carcinogenic
HTnc: human toxicity: non-carcinogenic
IR: ionizing radiation
LU: land use
LCA: life cycle assessment
LCI: life cycle inventory
LCIA: life cycle impact assessment
ME: marine eutrophication
MET: marine ecotoxicity
MRS: mineral resource scarcity
SOD: stratospheric ozone depletion
FPM: fine particulate matter formation
TA: terrestrial acidification
TET: terrestrial ecotoxicity
WC: water consumption
