# Supplementary material for: Life Cycle Assessment of Microbial 2,3-Butanediol Production from Brewer’s Spent Grain Modeled on Pinch Technology
Source: ACS Sustain Chem Eng. 2023 May 22;11(22):8271–80. doi: 10.1021/acssuschemeng.3c00616 (PMC10245393; doi:10.1021/acssuschemeng.3c00616)
Supplement: Supplementary file 1 — sc3c00616_si_001.pdf [file sc3c00616_si_001.pdf]

Supporting Information

Life Cycle Assessment of Microbial 2,3-Butanediol Production from Brewer's Spent Grain Modeled on Pinch Technology

Bikash Ranjan Tiwari,[†] Rajarshi Bhar,[‡] Brajesh Kumar Dubey,[‡] Sunil K. Maity,[§] Satinder Kaur Brar,[*,∥] Gopalakrishnan Kumar[⊥] and Vinod Kumar [*,#,△]

[†]Institut national de la recherche scientifique - Centre Eau Terre Environnement, Université du Québec, Quebec City-G1K9A9, Canada

[‡]Department of Civil Engineering, Indian Institute of Technology Kharagpur, West Bengal-721302, India

[§]Department of Chemical Engineering, Indian Institute of Technology Hyderabad, Kandi, Sangareddy 502284 Telangana, India

[∥]Department of Civil Engineering, Lassonde School of Engineering, York University, North York, Toronto-M3J1P3, Canada

[⊥]School of Civil and Environmental Engineering, Yonsei University, Seoul 03722, Republic of Korea

[#]School of Water, Energy and Environment, Cranfield University, Cranfield MK43 0AL, United Kingdom

[△]Department of Biosciences and Bioengineering, Indian Institute of Technology Roorkee, Roorkee 247667, Uttarakhand, India

*Corresponding authors

Email address: Vinod.Kumar@cranfield.ac.uk, Satinder.Brar@lassonde.yorku.ca

Number of pages: 2

Number of tables:1

Number of figures: 0

Table S1: Inventory for background processes used in life cycle analysis

Components	Library
Water	Water, deionised {RoW}| water production, deionised | Cut-off, U
Glucose	Glucose {RER}| glucose production | Cut-off, U
Yeast extract	Protein feed, 100% crude {GLO}| fodder yeast to generic market for protein feed | Cut-off, U
Phosphoric acid	Phosphoric acid, industrial grade, without water, in 85% solution state {RER}| purification of wet-process phosphoric acid to industrial grade, product in 85% solution state | Cut-off, U
Potassium hydroxide	Potassium hydroxide {RER}| production | Cut-off, U
Magnesium sulfate	Magnesium sulfate {RER}| production | Cut-off, U
Acetic acid	Acetic acid, without water, in 98% solution state {RER}| acetic acid production, product in 98% solution state | Cut-off, U
Sodium hydroxide	Sodium hydroxide, without water, in 50% solution state {RER}| chlor-alkali electrolysis, mercury cell | Cut-off, U
Manganese sulfate	Manganese sulfate {GLO}| production | Cut-off, U
Transportation	Transport, freight, lorry 16-32 metric ton, EURO5 {RER}| transport, freight, lorry 16-32 metric ton, EURO5 | Cut-off, U
Sulfuric acid	Sulfuric acid {RER}| production | Cut-off, U
Enzyme	Enzyme, Cellulase, Novozyme Celluclast/kg/RER
Peptone	Chemical, organic {GLO}| production | Cut-off, U
Manure spreading	Solid manure loading and spreading, by hydraulic loader and spreader {RoW}| processing | Cut-off, U
Electricity	Electricity, medium voltage {GB}| electricity voltage transformation from high to medium voltage | Cut-off, U
Wastewater treatment	Wastewater, unpolluted {RoW}| treatment of, capacity 5E9l/year | Cut-off, U
N-fertilizer	Ammonium nitrate, as N {RER}| ammonium nitrate production | Cut-off, U
P-fertilizer	Phosphate fertiliser, as P_2O_5 {RER}| triple superphosphate production | Cut-off, U
K-fertilizer	Potassium chloride, as K_2O {RER}| potassium chloride production | Cut-off, U
